# Work related injury among Saudi Star Agro Industry workers in Gambella region, Ethiopia; a cross-sectional study

**DOI:** 10.1186/s12995-017-0153-x

**Published:** 2017-03-16

**Authors:** Daniel Haile Chercos, Demeke Berhanu

**Affiliations:** Department of Environmental and Occupational Health and Safety, Institute of Public Health, University of Gondar, P.O. Box 196, Gondar, Ethiopia

**Keywords:** Agro industry, Ethiopia, Work related injury, Working environment

## Abstract

**Background:**

Work injury is an important cause of morbidity and mortality, much of these work injuries burden can be found in industry required heavy manual work such as, agriculture and fishers. Hence; agriculture is consistently cited as one of the most hazardous industry in the world. The objective of this study isto assess the magnitude and associated factors of work related injury among Saudi Star Agro Industry workers in Gambella region, South West Ethiopia.

**Methods:**

An institutional based cross-sectional study design was conducted on Saudi Star Agro Industry located in Gambella region, from February - June 2014 on 449 randomly selected workers who arestratifiedby working department. Anobservation checklist, factory clinical records and a structured interview questioner were used as a data collection tools.

**Result:**

The prevalence of work related injury was 36.7%. Marital status [AOR;1.69, 95%; CI;(1.1–2.7)], service year [AOR;1.9,95%; CI;(1.17–3.1)], working more than 48 h per week [AOR;9.87, 95%; CI;(5.95–16.28)],safety training [AOR;3.38, 95%;CI;1.14–9.98)], regular health checkup [AOR; 12.29, 95%; CI (9–51.35)] and usage of personal protective equipment [AOR; 2.36, 95%; CI; (1.06–5.25)] were significant factors for the occurrence of work related injury.

**Conclusion:**

The prevalence of work related injury was high. Working hours, safety training and regular health checkup increases the risk of work related injury.

## Background

Occupational injuries are one of the major public health problems in the world. This is because the total consequence of occupational injury extend well beyond direct physical injury and include a wide array of social and economic burdens [1]. Work related injury is an important cause of morbidity and mortality and much of work related injury burden can be found in industries requiring heavy manual work such as agriculture and fishers [2–4]. However most of work related injuries can be prevented by using appropriate occupational safety and health service as well as by using ongoing injury surveillance [5].

Globally, the burden of occupational injury accounts for 100 million cases per year, in which 360,000 are fatal accidents [6]. Reports showed that Developing countries have the highest injury fatality rate, in which 14 death reported per 100,000 workers due to occupational injuries [6, 7]. This results a loss of about 4% of world Gross National Product and the impact is estimated to be 10–20 times more in developing countries [8].

Agriculture is consistently cited as one of the most hazardous industry in the world. As a result, workers and their families are vulnerable to high injury and fatality rates. In 2008, the farmer and rancher occupation had a fatality rate that was 10 times more when compared with all occupations (40.3 Vs 3.7 per 100,000 workers) [5]. This indicates that workers in the agriculture sector suffer from higher rates of accidents and fatal injuries than workers found in other industries [3, 4].

Agriculture, have many organizational and environmental characteristics that can affect the health of workers including; exposure to hazardous conditions in the natural environment, use of dangerous machinery and chemicals and unconventional work arrangement [2–4]. On top of that, the introduction of new technologies and new chemical substances have led to new occupational injuries [8].

Agriculture constitutes the major economical share for most of the Sub-Saharan countries including Ethiopia. In Ethiopia, the number of industries in the agricultural sector is increasing recently and it became the backbone of the Ethiopian economy. In Ethiopia; agricultural industries accounts for almost all of the foreign exchange earnings of the country and it provides almost 50% of the country’s Growth Domestic Product (GDP), with nearly 80% of the labor force working in this sector [9]. Although there are few studies done so far that had made considerable progress in protecting workers from occupational injury and illness, there are still vast unreported occupational accidents and diseases exists [10]. In addition, a geographical variation is a big factor for fatality rate of occupational injuries. Therefore it is important to study and take preventive measure related to work related injuries in agricultural sector of a country [11].

## Methods

### Aim of this study

The aim of this study is to assess the prevalence and associated factors of work related injury among Saudi Star Agro Industry workers, Gambella region, South West Ethiopia.

### Study design

An institutional based cross-sectional study design was conducted from February - June 2014.

### Study area and period

This study was conducted at Saudi Stare Agro Industry. The study area is located in South West of Ethiopia, Gambella region, Aboboworeda, which is about 813 km from the capital city, Addis Ababa. The company was established in 2007 and has a current production capacity of 12800 t of rice per year. Currently, the industry has employed 1064 workers from which 244 (22.94%) are female workers.

### Source population

All workers who were working in agricultural production segment of Saudi Stare Agro Industry.

### Inclusion and exclusion criteria

#### Inclusion criteria

All agricultural workers involved in agricultural production segment.

#### Exclusion criteria

Administrative staffs, workers on annual leave and workers who were absence during data collection period.

### Sample size

The sample size was calculated using a single population proportion formula. It was calculated taking 95% confidence interval, marginal error 4%, and work related injury as 78.3% [12]; yielding a sample size of 449 workers.

Three departments were selected as the major area of the enterprise where workers directly involve in agricultural division. Assuming that work related injury varies with the nature of the work; the calculated sample size was distributed across the selected three departments using stratified sampling technique. Study subjects were allocated proportionally from each department and finally subjects were drawn by simple random sampling technique from each department sampling frame.

### Operational definitions

- Work related injury; any physical injury condition sustained on worker in connection with the performance of his/her work but not include work related diseases that need exposure assessment and laboratory tests [13].
- Personal protective equipment (PPE); Utilization of the worker specialized clothing or equipment worn by employees for protection against health and safety hazards at the time of interview [14].
- Manual handling; the movement or support of any load effort, including; lifting put down pushing, puling, carrying and moving.
- Sleeping disorder; the presence of sleeping problems when the workers are at work in the factory [14].
- Safety guarding of machine; the machine is safe if it safe guards workers from contacts with dangerous moving parts [7].
- Agricultural injury; is defined as unintentional physical injury or poisoning which occurred during an agricultural activities and which required medical attention [5].
- Incident; any unplanned event resulting in, or having the potential for injury, illness, in health, damage or other loss [5].
- Excessive heat: heat is recorded as excessive if a worker is found sweating when naked or with light clothing; if the investigator feels a sudden heat wave when entering to the work [7].

### Data collection tools and procedures

The data was collected using face to face interview administered from pre – tested structured questionnaire developed from International Labor Organization (ILO), Occupational Safety and Health (OSH) policy 2012 standards and other studies modified for the purpose of this study. Observations were also made by principal investigator using prepared observational checklist to evaluate work environment. Moreover, record reviews from clinic and safety committee group discussion were also used as assertion to the self-reported information made by study respondents. Seven data collector, one supervisor and one principal investigator were enrolled during data collection.

### Data quality control

The questionnaire was developed first in English and translated to Amharic and back to English by language experts for consistency validity. The data collectors were trained for 3 days about data collection tool, questioning technique and ethical issues. A pre-test was also conducted on similar industry to assess the validity and reliability of the questionnaire. The completeness of the questionnaires was checked before data entry.

### Data processing and analysis

The data was entered in Statistical Package for Social Sciences (SPSS) 16 for analysis. All assumptions applied to binary regression including fitness of model were checked. The findings were present by using frequencies, tables, and graphs. The presence of interaction between independent factors explored. To identify factors associated with work related injury, Binary Logistic regression model was fitted and variables with a *p* < 0.2 in bivariate analysis included in the multi-variant analysis. Those predictors with *p*-value < 0.05, in the multi-variant analysis was considered as independent and significant predictors for work related injury and Odds ratio (OR) with 95% confidence interval was reported.

## Results

### Socio-demographic factors

Majority of a study participants, 265 (59%) were male. The minimum and maximum age was 18 and 41 respectively and 232 (51.6%) of respondent were single.

From a total of 449 respondent 152 (33.9%) were primary school (1–8 grade) and 269 (59.9%) respondents had 3 years and below service year experience. Regarding employment pattern, 413 (92%) were temporarily employed and 382 (85.1%) of the respondent were earned less than 1600 ETB (Table 1).

**Table 1:** Distribution of socio-demographic characteristics of respondents in Saudi Star Agro Industry in Gambelia region, Ethiopia, 2014 (*n* = 449)

Variable	Frequency(n)	Percent (%)
Sex		
Male	265	59%
Female	184	41%
Age		
18–29	324	72.2%
> 29	125	27.8%
Educational status		
Illiterate	10	2.2%
Read and write	132	29.4%
Primary school(1–8)	152	33.9%
Secondary school (9–12)	114	25.4%
TVT	37	8.2%
First degree and above	4	0.9%
Marital status		
Married	215	47.9%
Single	232	51.7%
Divorce	2	0.4%
Employment type		
Temporary	413	92%
Permanent	36	8%
Monthly income		
≤ 1600	382	85.1%
> 1600	67	14 .9%
Working experience		
≤ 3	269	59.9%
> 3	180	40.1%

### Work related injury characteristics

#### Prevalence of work related injuries

**Fig. 1:**
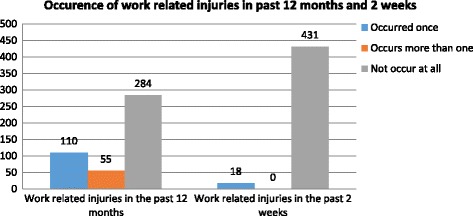
The prevalence and frequency of work related injuries in the past 12 months and 2 weeks among Saudi Star Agro Industry workers in Gambella region, Ethiopia, 2014

Among the study participants, 165 (36.7%) had work related injury in the last 12 months with the overall prevalence rate of 367 per 1000 exposed worker per year. Moreover, 18 (4%) respondent were also injured at job in the last 2 weeks. With respect to the frequency of injury occurrence in the last 12 months, 110 (24.5%) respondents were injured once, and 55 (12.2%) injured more than once (Fig. 1).

#### Cause, type and affected body part

From Injured respondents, predominantly affected parts of the body were; hand 46 (27.9%), leg 34 (20.6%), eye 25 (15.15%) and toe 23 (13.93%) (Table 2). With respect to type of injuries, 61 (37.0%) laceration, 25 (15.15%) eye injury, 24 (14.54%) cut and 23 (13.94%) puncture were the most type of injury reported by respondents (Table 3). Hand tool 63 (38.20%), machine 32 (20%), splinting objects 26 (15.75%) and lifting objects 17 (10.3%) were the top sources of work related injures (Table 4).

**Table 2:** Parts of the body injured and types of injury among workers in Saudi Star Agro Industry in Gambelia region, Ethiopia, 2014(*n* = 165)

Part of the body affected	Frequency	Percent (%)
Hand	46	27.9%
Toe	23	13.9%
Back	17	10.3%
Eye	25	15.2%
Finger	2	1.2%
Leg	34	20.6%
Ear	2	1.2%
Chest	8	4.8%
Upper arm	1	0.6%
Other	7	4.3%

**Table 3:** Type OF INJURES among injured worker in Saudi Star Agro Industry in Gambelia region, Ethiopia, 2014 (*n* = 165)

Types of injury	Frequency	Percent (%)
Abrasion/Laceration	61	37.0%
Cut	24	14.5%
Puncture	23	13.9%
Back pain	14	8.5%
Eye injury	25	15.2%
Ear injury	2	1.2%
Others	16	9.7%

**Table 4:** Source of injury among injured workers in Saudi Star Agro Industry in Gambelia region, Ethiopia, 2014 (*n* = 165)

Source of injury	Frequency	Percent (%)
Machine	33	20%
Falling object	15	9.1%
Splinting object	26	15.8%
Collision	2	1.2%
Acid and acidic substance	8	4.8%
Hand tool	63	38.2%
Lifting object	17	10.3%
Other	1	0.6%

**Fig. 2:**
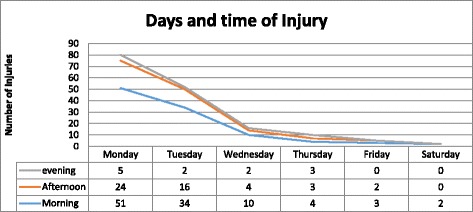
Days and time of work related injuries among Saudi Star Agro Industry workers in Gambella region, Ethiopia, 2014

With regard to the specific days of injuries, 80 (48.48%) were on Monday, 52 (31.51%) were on Tuesday and most respondent injured in the morning at the time of 6A.M – 6P.M (Fig. 2). When we see Absenteeism due to work related injuries in the industry in the last 12 months, 75 (16.7%) absenteeism occurred for 1 day, 10 (2.2%) for 2 days and 3 (0.2%) for 3 days.

Of those injured respondents 101 (61.2%) workers injured while in production department (cultivating, irrigation, and loading unloading) and 36 (21.8%) were injured in agro mechanization department (mechanic, tractor operator, loader operator and welder). The most frequent reason given by the respondents for the occurrence of injury were working behavior 79 (47.87%).

#### Severity of work related injury

Out of 165 injured respondents six (1.3%) were hospitalized and 87 working days were lost as the result of work related injury.

#### Description of work environment

**Fig. 3:**
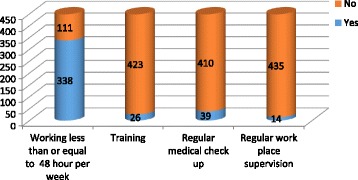
Work environment related factors among workers in Saudi Star Agro Industry in Gambella region, Ethiopia, 2014

Concerning with working hour, 338 (75.27%) respondents were working for 48 h per week and 111 (24.8%) were working for more than 48 h. From all respondents, 94.2% realized that they have no safety training and 96.88% of the respondents responded lack of supervision at work place (Fig. 3).

#### Behavioral characteristics

Among 449 respondents, 59 (13%) were drinking alcohol, 20 (4.5%) were chewing chat, 18 (4%) were smoking cigarette and 42 (4.5%) had sleep disorder. From all respondents 376 (83.75) were not using PPE. Reasons given by respondents for not using PPE are 307 (81.64%) were no PPE, 26 (6.91%) were lack of awareness, 10 (2.65%) don’t know how to use the PPE, 15 (3.98%) not comfortable and 18 (4.8%) were due to decrease performance of PPE.

#### Observation of work environment

During the observation, we have seen that most working section were with excessive heat and dust. In addition, there were no safety division and personnel in the enterprise that help in promoting health and safety condition at work place. Warning sign and health and safety instructions or procedure did not exist in all working section; similarly all working section lacks first aid equipment except they had clinic at central level (Table 5).

**Table 5:** Occupational health and safety hazards identified in working section, Saudi Star Agro Industry in Gambelia region, Ethiopia, 2014

Type of workplace	Work department	Hazard Identified
Agro mechanization	Farm mechanization	Excessive heat, dust, sharps, gasoline and Sulfuric acid
	Work shop/Garage	Excessive heat, sharps, gasoline and Sulfuric acid
Workshop/Garage	Pest control department	Pesticide and chemicals, and PPE are not Standardized
Others	Seed preparation and rice packing	Noise, Poor ventilation, sharps, dust, and have loaded materials
	Unit farm Irrigation	Excessive heat and dust, Snack bits Dust, and sharps

#### Associated factors of work related injury

From the socio-demographic variables, marital status and service years of workers showed significant association with work related injury. Workers who are single were 1.73 times more likely to report work related injury than workers who are in marriage [AOR; 1.73; 95%; CI (1.09–2.75) and workers whose service year less than or equal to 3 were 1.89 times more likely to report work related injury than whose service year above 3 years [AOR; 1.89; 95%; CI; (1.16–3.08). However sex, age, educational status, income and type of employment have no significance association with work related injury in this study.

Among work environment variables, hours worked per week, safety training and regular health checkup showed significant association with work related injury. Workers who worked more than 48 h per week were 8.33 times more likely to be injured than workers who spend their time in the work place for 48 h and less [AOR; 8.33; 95%; CI (4.87–14.41)]. Similarly, workers without safety and health training were 4.56 times more likely to be injured than who had training [AOR; 4.56; 95%; CI;(1.299–16.1)].In addition, workers who had no regular health checkup were 5.84 times more likely to be injured than who had regular health checkup [AOR; 5.56; 95%; CI (2.04–16.73)]. However, supervision of work place had no significant association with work related injury.

Among behavioral factors, usage of personal protective equipment was significantly associated with work related injury. Workers who did not used personal protective equipment’s were 2.58 times more likely to reported work related injury than workers who did use PPE in the work place [AOR; 2.58, 95%; CI (1.17–5.68]. However, smoking cigarettes, drinking alcohol, sleep disorder and chewing chat were not significantly associated with work related injury (Table 6).

**Table 6:** Summery of logistic regression analysis of the relative effect of work related injuries among SAUDI STAR AGRO INDUSTRY IN GAMBELLA REGION, ETHIOPIA, 2014

Characteristics	WRI		COR 95% CI	AOR 95% CI
	Yes	No		
Sex				
Male	86	176	0.719(0.49–1.06)^x^	0.63(0.4–1.01)^0^
Female	76	108	1.00	
Age				
≤ 29	126	198	1.04(0.9–2.18)^0^	1.28(0.76–2.17)^0^
> 29	39	86	1.00	
Marital status				
Single	107	127	2.28(1.54–3.39)^xxx^	1.6 (1.01–2.57)^x^
Married	58	157	1.00	1.00
Service				
≤ 3	123	146	2.77(1.8–4.22)^xxx^	2.05(1.22–3.29)^xx^
> 3	42	138	1.00	1.00
Safety				
No	161	262	3.38(1.14–9.98)^x^	4.89(1.37–17.4)^x^
Yes	4	22	1.00	1.00
PPE				
No	155	221	4.42(2.19_8.89)^xxx^	2.54(1.15–5.64)^x^
Yes	10	63	1.00	1.00
Health checkup				
No	161	248	5.84(2.04–16.73)^xx^	4.06(1.1–14.99)^x^
Yes	4	36	1.00	1.00
Chewing chat				
No	5	15	0.55(0.2–1.57)^0^	0.66(0.14–3.04)^0^
Yes	160	269	1.00	
Working hour				
≤ 48	81	257	1.00	1.00
> 48	84	27	9.87(5.98–16.28)^xxx^	8.53(4.9–14.73)^xxx^

^0^NB variable whose *P*-value < 0.3 in bivariate; ^x^significant at *P* < 0.05; ^xx^significant at *P* < 0.01; ^xxx^significant at *P* < 0.001

## Discussion

### Magnitude and severity of work related injury

Determining the prevalence of work related injuries and identifying associated factors are essential in the development of injury prevention strategy at the work place. The overall prevalence of work related injury in this study was 36.7% or 367 per 1000 workers per year. This finding is similar with studies done in agricultural workers stating that workers suffer markedly high rate of injuries than any other workers [5, 15]. In addition, most workers in this study are temporary workers (92%) and temporary workers and daily laborers are among the most vulnerable groups in agricultural workplaces [16].

This study indicates high rate of injury compared to a study made on other industries [2, 17, 18]. This could be due to poor promotion and preventive work related to safety and health such as, lack of safety training, lack of regular health checkup, lack of poor usage of personal protective equipment’s, and being temporary worker (temporary workers does not get equal benefits with permanent workers in most industries) may contribute to high rate of injury in this study.

### Major work related injury types, part of the body affected, and source of injury

The finding of this study indicates that, abrasion/laceration, eye injury, cut and punctures as the most frequent types of injuries. These findings are consistent with studies conducted in Ethiopia; study conducted in Tendaho agricultural industry and a study done in Gondar on small and medium scale industries [8, 18]. These findings are also consistent with a study done on risk of agricultural injury among African-American farm works from Alabama and Mississippi and a study conducted on eye health and safety among Latino farm workers [19, 20]. In addition literature revealed that the stated findings are common in work related injury [3, 21].

This study also revealed that hand tools, machine and splinting objects are the common source of injury. This finding is consistence with a study done in agricultural injury in rural California [21]. The reason for this may be due to the fact that most of the workers were temporary and daily laborer. These workers are characterized by manual handling and working on environment which is full of pieces of stone and dry soil. The risk of temporary workers and manual worker as the most exposed group in agricultural activities is well documented in study done in Easter U.S [22]. These findings are also in agreement with other studies done in Ethiopia such as Tendho agricultural industry, large scale metal manufacturing industry in Addis Ababa, and on small and medium scale industry in Gondar [7, 8, 18].

In this study we have found that hand, leg, eye and toe were the most common parts of the body injured. This finding is in agreement with the study done in Tendaho agricultural industry in Afar, Ethiopiaand a study done on farm related injury on older Kentucky farmers in U.S. [8, 23].

### Determinant of work related injury

The finding of the studies revealed several factors that related to the occurrence, severity and types of injury. Among the assessed socio-demographic determinant of work related injury marital status and service year were significantly associated with work related injury. This finding is consistent with the study done in Tendho agricultural industry, study done on large scale manufacture industries in Addis Ababa and study done on small and medium scale enterprise in Gondar [7, 8, 17]. In addition, this finding is also consistent with studies done on Kentucky farmers [18].

The finding of this study showed that from all work environmental factors, working hour per week, safety training and regular health checkup were significantly associated with work related injury. These are in agreement with studies done in Tendho manufacturing industry, large manufacturing industries in Addis Ababa, a study done in small and medium enterprise in Gondar, Ethiopia and a study done on occupational injuries in Kombolcha textile factory, Ethiopia [7, 8, 13, 18]. Similarly, the finding of the study is consistent with the studies done on fatal occupational injury among non-governmental employee in Malaysia and injuries and fatalities on older farmers in U.S. [3, 22].

Among the assessed behavioral determinant of work related injury, using of personal protective equipment was significantly associated with injury. This finding is in agreement with studies done in large manufacturing industry in Addis Ababa, study done in Tendho agricultural industry in Afar, study done on small and medium enterprise in Gondar, and study done on occupational injuries among Addis Ababa city municipal solid waste collectors [7, 8, 18, 24]. Similar finding is also observed on studies done on injuries and fatalities in U.S farmers and agricultural injuries on older farmers in Kentucky [22, 23].

## Conclusion

The prevalence of work related injury in Saudi Star Agro Industry was high. Marital status, service year, usage of personal protective equipment, safety training regular health checkup and working hours per week were significantly associated with work related injuries.

## Abbreviations

AOR: Adjusted odds ratio
CI: Confidence interval
GDP: Growth domestic product
ILO: International Labor Organization
IRB: Institutional Review Board
OR: Odds ratio
OSH: Occupational safety and health
PPE: Personal protective equipment
SPSS: Statistical package for social sciences
